# Synaptic Pruning by Microglia in Epilepsy

**DOI:** 10.3390/jcm8122170

**Published:** 2019-12-09

**Authors:** Megumi Andoh, Yuji Ikegaya, Ryuta Koyama

**Affiliations:** Laboratory of Chemical Pharmacology, Graduate School of Pharmaceutical Sciences, The University of Tokyo, 7-3-1 Hongo, Bunkyo-ku, Tokyo 113-0033, Japan; 21100750megumi@gmail.com (M.A.); yuji@ikegaya.jp (Y.I.)

**Keywords:** microglia, synapse, E/I balance, engulfment, synaptic pruning, epilepsy, seizure, C1q

## Abstract

Structural and functional collapse of the balance between excitatory (E) and inhibitory (I) synapses, i.e., synaptic E/I balance, underlies the pathogeneses of various central nervous system (CNS) disorders. In epilepsy, the synaptic E/I balance tips toward excitation; thus, most of the existing epileptic remedies have focused on how to directly suppress the activity of neurons. However, because as many as 30% of patients with epilepsy are drug resistant, the discovery of new therapeutic targets is strongly desired. Recently, the roles of glial cells in epilepsy have gained attention because glial cells manipulate synaptic structures and functions in addition to supporting neuronal survival and growth. Among glial cells, microglia, which are brain-resident immune cells, have been shown to mediate inflammation, neuronal death and aberrant neurogenesis after epileptic seizures. However, few studies have investigated the involvement of synaptic pruning—one of the most important roles of microglia—in the epileptic brain. In this review, we propose and discuss the hypothesis that synaptic pruning by microglia is enhanced in the epileptic brain, drawing upon the findings of previous studies. We further discuss the possibility that aberrant synaptic pruning by microglia induces synaptic E/I imbalance, promoting the development and aggravation of epilepsy.

## 1. Introduction

Because the synchronized hyperactivity of neurons shapes epilepsy, researchers and clinicians have focused their efforts on understanding the functions of neurons (both excitatory and inhibitory), pinning their hopes for therapeutic targets on those related to neuronal firing. However, as many as 30% of patients with epilepsy continue to struggle with drug-resistant seizures, suggesting a frontier for the development of novel therapeutic strategies for epilepsy other than merely manipulating the activity of neurons [1,2]. We propose glial cells, which are no longer believed to be just ‘glue’ for neurons, as promising next-generation targets for intractable epilepsy. Glial cells dominate the brain in number, exhibiting populations ten times as high as those of neurons, and are often upstream modulators of neuronal survival and functions, including neuronal activity. Indeed, glial cells such as microglia and astrocytes have been the subjects of pioneering studies on epilepsy, and some studies have discovered a link between glial function and functional imbalance of excitatory versus inhibitory synapses. For decades, the hypothesis that synaptic excitatory/inhibitory (E/I) balance is disturbed toward the increased excitation of synaptic circuits in the epileptic brain has been supported. However, this hypothesis has been challenged by findings that some types of seizures can be induced when inhibitory transmission is enhanced. For example, the tonic GABA_A_ currents of thalamocortical neurons are increased in gamma-hydroxybutyric acid (GHB, a GABA_B_R agonist)-administered rats and GABA transporter (GAT-1)-knockout mice—both of which are animal models of absence epilepsy [3]. Furthermore, some gene mutations related to epilepsy, such as mutations in syntaxin binding protein 1 (*STXBP1*, the risk gene for Ohtahara syndrome) and protocadherin 19 (*PCDH19*, the risk gene for multiple types of seizures), do not induce synaptic E/I imbalance [4]. However, it is possible that synaptic E/I balance might be impaired during or shortly before and after epileptic seizures. For example, in a rat model of temporal lobe epilepsy which was induced by intrahippocampal injection of tetanus neurotoxin (TeNT), hippocampal synaptic E/I balance tips toward excitation and intermittent spontaneous seizures were observed 8–16 days after TeNT injection [5]. Synaptic E/I imbalance has also been suggested to be involved in other diseases. For example, patients with Alzheimer’s disease (AD) sometimes exhibit seizure-like electroencephalogram (EEG) signals even before the onset of cognitive impairments [6]. Seizure-like EEG signals have also been confirmed to occur in the cerebral cortex and hippocampus in AD model mice [7]. Furthermore, neuronal activity is increased in animal models of multiple sclerosis (MS), Parkinson’s disease (PD) and autism spectrum disorders (ASDs), brain diseases that are sometimes accompanied by epileptic seizures [8,9,10,11,12]. Thus, it is likely that synaptic E/I imbalance is the main cause of epilepsy, but whether glial cells are involved has not yet been clarified.

The shared pathological feature of these neurological disorders is synaptic degeneration. Accumulating research has shown that abnormalities in synaptic number and function underlie synaptic E/I imbalance [13]. Because both synaptic number and function can be regulated by glial cells, including microglia and astrocytes [14,15,16], it is possible that these glial cells play crucial roles in the pathogeneses of neurological disorders. Microglia are brain-resident immune cells that patrol the brain and control synaptic number either by pruning synapses or by promoting the formation of synapses [17,18]. On the other hand, astrocytes, which form the largest population among glial cell types, can control synaptic activity by taking up neurotransmitters in the extrasynaptic space [15]. Dysfunction of astrocytes in the epileptic brain has been well studied, but whether synaptic pruning by microglia plays a role in epilepsy remains largely undetermined [19]. In this review, we discuss the possibility that aberrant synapse engulfment by microglia contributes to the pathogenesis and deterioration of epilepsy by causing synaptic E/I imbalance.

## 2. Changes in Microglial Properties in the Epileptic Brain

### 2.1. Morphological Changes

**Figure 1:**
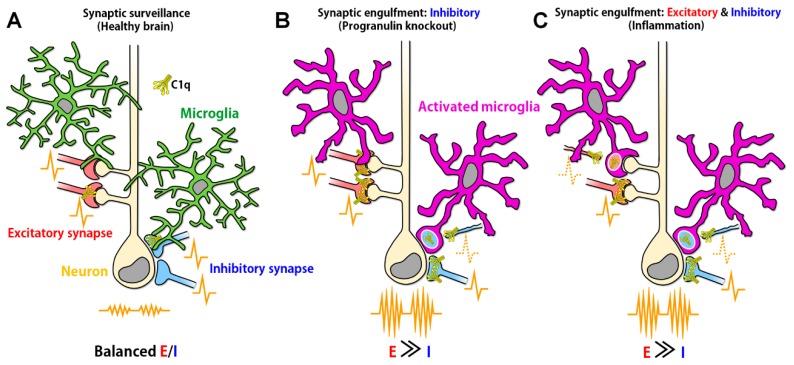
Aberrant synaptic engulfment by microglia possibly induces synaptic excitatory/inhibitory (E/I) imbalance. (**A**) In the healthy brain, in which synaptic E/I balance is properly maintained, ramified microglia continuously move their processes, surveying neuronal synapses. (**B**) In the ventral thalamus of progranulin knockout mice, C1q expression was increased and activated microglia-engulfed inhibitory synapses, causing E/I imbalance (reference [25]). However, it remains unclear why microglia selectively engulfed inhibitory synapses even when both excitatory and inhibitory synapses were tagged by C1q. (**C**) In the hippocampus of West Nile virus-infected mice, C1q expression was increased and activated microglia possibly engulfed both excitatory and inhibitory presynaptic terminals (reference [26]) because the presynaptic markers used in the study are expressed in both excitatory and inhibitory synapses. Loss of excitatory and inhibitory synapses could result in E/I imbalance (reference [27,28]). However, the mechanism by which neuronal activity tipped toward excitation even when both excitatory and inhibitory presynaptic transmissions were decreased remains unrevealed. The engulfment of presynapses was also increased in the frontal cortex of systemic lupus erythematosus model mice (reference [29]), implying the possible E/I imbalance in this model mice.

Microglia generally possess highly branched processes and exhibit a ‘ramified’ shape in the brain, surveying synaptic structure and function (Figure 1A). In patients with mesial temporal lobe epilepsy (mTLE) and hippocampal sclerosis, who have experienced recurrent seizures, hippocampal microglia exhibit an ameboid shape, which is the typical morphological sign of microglial activation [20]. Ameboid microglia have also been observed in the hippocampus in a mouse model 24 to 48 h after kainic acid-induced status epilepticus [21]. On the other hand, the number of microglial processes increases, and the morphology of microglia becomes hyperramified 3 to 8 h after kainic acid-induced status epilepticus [22]. The microglial number and morphology were not altered 55 days after injection of TeNT into primary visual cortex of mice, though electrographic epileptiform activity was observed [23]. However, because the dendritic spine density was decreased at this period, it is possible that enhanced synaptic engulfment by microglia contributed to the decreased spine density in this model [24]. The TeNT-induced seizure model is useful to directly study synaptic E/I imbalance in epilepsy without genetic mutations, but we need future studies to further examine microglial involvement in synaptic E/I imbalance. As microglial morphology and their functions, especially surveillance of the brain environment, are closely related, the above findings may reflect changing roles of microglia after seizure onset that depend on the phase and severity of the seizures.

### 2.2. Molecular Expression

After epileptic seizures, microglial expression of inflammatory cytokines, purinergic receptors and fractalkine receptors significantly increases. The fractalkine receptor CX3CR1 is mainly expressed by microglia, and its ligand CX3CL1 is expressed by neurons in the brain parenchyma. CX3CL1–CX3CR1 signaling mediates various types of process involving microglia–neuron interactions, such as synaptic pruning, synaptic plasticity and adult neurogenesis in the hippocampus [30,31,32]. In the hippocampus and temporal neocortex in mTLE patients, immunohistochemical and Western blot analyses have revealed that CX3CL1 levels are increased, and the protein levels of CX3CL1 in cerebrospinal fluid and serum of patients have also been shown to be increased by enzyme-linked immunosorbent assay (ELISA) [33]. Elevated CX3CL1 immunoreactivity has also been confirmed in the hippocampus 1 to 3 h after pilocarpine-induced status epilepticus in rats [34]. The increased CX3CL1 levels decrease to control levels 3 days after status epilepticus. In contrast, CX3CR1 immunoreactivity in pilocarpine-treated rats remains higher than that in control rats 3 days after status epilepticus [34]. Therefore, it is still under debate whether CX3CL1–CX3CR1 signaling is enhanced in the epileptic brain.

Microglia express several types of ATP receptors, such as P2X4, P2X7, P2Y4, P2Y6, P2Y7 and P2Y12 [35,36]. In the hippocampus in a kainic acid-induced mouse model of mTLE, P2Y6 mRNA levels were increased 3 h after status epilepticus, and P2Y4, P2Y6, P2Y7 and P2Y12 mRNA levels were also increased 24 to 48 h after status epilepticus [21]. Microglial P2X7 immunoreactivity is elevated in the rat hippocampus 24 h after kainic acid-induced seizures [22]. P2X7 activation promotes IL-1β processing and tumor necrosis factor-α (TNF-α) expression—both of which enhance neuronal hyperexcitability. However, whether P2X7 has proconvulsive or anticonvulsive effects that vary according to the animal models of status epilepticus: P2X7 plays proconvulsive roles in pilocarpine-induced status epilepticus and anticonvulsive roles in status epilepticus triggered by intra-amygdala injection of kainic acid [37,38]. Furthermore, P2X4 levels are increased in mouse hippocampal microglia after kainic acid-induced status epilepticus. P2X4-positive microglia have been found to enhance neuronal activity through downregulation of the chloride-potassium cotransporter KCC2 in a mouse model of neuropathic pain [39]. However, P2X4 deficiency in rats does not affect susceptibility to kainic acid-induced status epilepticus [22]. In the hippocampus in a kainic acid-induced mouse model of mTLE, P2Y6 mRNA levels were increased 3 h after status epilepticus, and P2Y4, P2Y6, P2Y7 and P2Y12 mRNA levels were also increased 24 to 48 h after status epilepticus [21]. As elevations in P2Y6 after the administration of kainic acid increase the phagocytosis of microspheres by microglia in vitro and in vivo, P2Y6 could mediate the phagocytosis of synapses or dead neurons after seizures [40]. However, the involvement of microglial P2Y4 and P2Y7 in epileptic pathophysiology has not been revealed. Because activation of microglial P2Y12 promotes the elongation of microglial processes, as mentioned in Section 5.1.1 below, it is possible that microglia–neuron interactions increase after epileptic seizures [41].

Microglia are referred to as brain-resident immune cells partly because they are capable of producing and releasing various cytokines. In the hippocampal CA1 region in a kainic acid-induced mouse model of mTLE, microglial expression of the lysosomal proteases cathepsin B, D and S was found to be increased, which implies that the phagocytic ability of microglia is enhanced [42,43]. Microglial TGF-β1 mRNA expression has also been found to be elevated in the hippocampus and temporal lobe in a kainic acid-induced rat model of mTLEs [44]. Because TGF-β1 has been reported to suppress the microglial inflammatory response, the increased TGF-β1 in microglia after epileptic seizures might act in a negative feedback system to prevent overactivation of microglia [45,46,47]. Microglial IL-1β mRNA expression was also found to be increased in the hippocampus and amygdala in a kainic acid-induced rat model of mTLE [48]. In a pilocarpine-induced mouse model of status epilepticus, microglial TNF-α mRNA was increased in various brain regions, including the hippocampus and cortex [49]. As the levels of both proinflammatory (IL-1β, IL-6 and TNF-α) and anti-inflammatory cytokines (IL-4, IL-10 and TGF-β1) are increased in microglia after epileptic seizures, we cannot conclude whether the microglial inflammatory response is enhanced or diminished in the epileptic brain [44,48,49,50].

### 2.3. Neurodegeneration

Both pilocarpine- and kainic acid-induced epileptic seizures cause neuronal death in the hippocampal CA1 and CA3; severe neuronal loss and gliosis in these brain regions are commonly reported together as hippocampal sclerosis in human temporal lobe epilepsy (TLE) patients [51,52]. Seizure-induced neurodegeneration is inhibited by the administration of minocycline, a tetracycline antibiotic that is often used to inhibit microglial activation, 1 day before pilocarpine-induced status epilepticus [51]. Minocycline has also been found to suppress neurodegeneration in the hippocampus in a kainic acid-induced mouse model of mTLE [53]. These results suggest that activated microglia promote neuronal death after epileptic seizures.

### 2.4. Neurogenesis

Aberrant neurogenesis in the hippocampus is observed after both pilocarpine- and kainic acid-induced status epilepticus and has been suggested to induce further epileptic seizures by forming ectopic neural circuits [54,55,56,57]. Under physiological conditions, microglia have been suggested to contribute to the proliferation, survival and differentiation of newborn neurons [58]. Inhibition of microglial activation by minocycline or a fractalkine receptor blocker decreases the number of newborn neurons after status epilepticus, suggesting that microglia may mediate excess neurogenesis in the epileptic brain [59,60]. In contrast, microglia suppress neurogenesis by engulfing ectopic newborn neurons in the dentate hilus after kainic acid-induced statue epilepticus in mice [61]. Furthermore, activation of microglial toll-like receptor 9 (TLR9) after epileptic seizures inhibits neurogenesis in kainic acid model mice [62]. Toll-like receptors (TLRs) activate innate immune responses through recognition of DNA and RNA derived from pathogens, and microglia express various types of TLRs in addition to TLR9 [63]. Thus, further studies on the roles of microglial TLRs in the epileptic brain should be conducted.

It is still debatable whether microglia promote or suppress neuronal death and neurogenesis after seizures. Thus, at this point, it cannot be determined whether microglia are neuroprotective or neurodegenerative in the epileptic brain. To clarify the role of microglia, researchers might also need to investigate the environment and cell types outside of the brain parenchyma. Recently, it has also been shown that the function of the blood-brain barrier (BBB) is disrupted in the context of epilepsy, which suggests that the cerebrovascular system might also affect microglial functions [64]. As almost all previous studies, both basic and clinical, have focused on changes in microglial properties after seizures, it remains largely unknown whether microglia contribute to epileptogenesis. To prevent the development of epilepsy, it is crucial to discover the cellular and molecular mechanisms underlying synaptic E/I imbalance during epileptogenesis. Even though epilepsy has been widely defined as a synaptopathy, and microglia are essential coordinators of synaptic circuits, no study has directly investigated microglia–synapse interactions in epilepsy.

## 3. Synaptic Pruning by Microglia

In the 1970s, it was shown that cortical synaptic density drastically increases before and after birth and decreases during childhood and adolescence to adult levels [65]. The reduction in synaptic number during development is called synapse elimination [17,66]. Elimination of unnecessary synapses and the subsequent morphological and functional maturation of uneliminated synapses are suggested to be crucial for the refinement of neuronal circuits and normal brain function. Indeed, excessive and insufficient densities of synapses are pathological features of autistic and schizophrenic brains, respectively [67].

Microglia, as brain-resident immune cells, exert phagocytic activity to engulf dead cells and pathogens and secrete inflammatory mediators. Recent studies have shown that microglia play important roles not only during inflammation but also in healthy brains [68]. One of these roles is synaptic pruning during development. It has been reported that synaptic pruning by microglia is regulated by classic complement molecules (C1q and C3) in the dorsal lateral geniculate nucleus (dLGN) in mice [17]. It has also been revealed that microglia preferentially engulf less active synapses in the dLGN [17]. Given these results, it is hypothesized that C1q is secreted from more active synapses and tags less active synapses, triggering a complement cascade in the less active synapses [69]. This process would allow microglia to recognize activated C3 in less active synapses through CR3 to support phagocytosis [69].

Recent studies have revealed that disruptions in synaptic pruning by microglia cause synaptopathies, including ASDs and schizophrenia [12,70]. Furthermore, it has been shown that microglia also engulf synapses in the brains of patients with AD, MS and PD [71,72,73]. Studies on patients and animal models of these disorders have revealed evidence of enhanced complement cascades, suggesting that complement-mediated synaptic pruning is promoted in these contexts.

## 4. The Complement System in the Epileptic Brain

The expression levels of complement molecules are increased in the brains of patients with epilepsy and in corresponding animal models. qPCR and Western blot analyses of postmortem brains have revealed that mTLE patients with hippocampal sclerosis have higher levels of C1q, C3 and iC3b (the cleaved form of C3) in the hippocampus than control patients [74]. While C1q colocalizes with microglia, astrocytes and neurons, the major sources of C3 are astrocytes and microglia. The mTLE patients with hippocampal sclerosis in this study had 16 seizures per month for 24 years on average. Thus, complements might be intermittently released from activated microglia and astrocytes and accumulate in the brain parenchyma. Interestingly, mTLE patients without hippocampal sclerosis do not show elevated expression of complement molecules. Because TLE with hippocampal sclerosis is prone to be intractable, the increases in complement molecules likely reflect the severity of epileptic pathophysiology. The same study also showed a positive correlation between the iC3b level and the number of spontaneous seizures after induction of status epilepticus in a kindling rat model. Another study investigated the expression of complement molecules and phagocytic receptors in the cortices of patients with refractory epilepsy caused by focal cortical dysplasia [75]. Western blot analysis indicated that there were higher levels of C1q and iC3b in the epileptic brains than in the control brains, and immunohistochemical analysis revealed that C1q was sandwiched between Iba1 (a marker for microglia) and microtubule associated protein 2 (MAP2, a marker for neurons), suggesting that the microglia–neuron interaction occurs via complement cascades. The increased expression of complements and the possible interaction between neurons and microglia implies the possible engulfment of neuronal subregions by microglia. Indeed, the expression levels of the phagocytic receptors triggering receptor expressed on myeloid cells 2 (Trem2) and Pros1 (a crosslinking molecule between the proto-oncogene MER tyrosine protein kinase (MerTK) and phosphatidylserine) were decreased, but MerTK expression was increased.

The expression patterns and levels of complement molecules have also been examined in animal models of epilepsy. In a pilocarpine-induced rat model of mTLE, an increased level of hippocampal C1q expression was detected at 2 weeks after the induction of status epilepticus [76]. With regard to the expression of complement receptors in microglia, the findings have varied by used animal species and the methods used to detect complements. In a kainic acid-induced mouse model of mTLE, the number of CR3-positive microglia was higher in the subiculum and entorhinal cortex in model mice than in control mice from 24 h to 3 months after induction of status epilepticus [77]. In another report, RT-qPCR conducted on hippocampal microglia 24 h after kainic acid injection showed that the expression levels of Trem2, MerTK, CR3 and probable G-protein coupled receptor 34 (GPR34) which are molecules related to phagocytosis, were decreased despite increased expression of purinergic receptors [78], which, when activated by ATP, can induce structural interaction between microglia and neurons (see Section 5.1).

Given the above evidence, it is likely that complement cascades, which are triggered by C1q, are enhanced in the epileptic brain, but the source of the released C1q is still unclear. Although neurons can secrete C1q in some brain regions during development, microglia are suggested to be the main source of C1q in aged mouse brains [79]. It has not been clarified what triggers the expression of C1q in epilepsy, but it is possible that microglia release C1q in response to neuronal hyperactivity, because both elevated neuronal activity and activated complement cascades have been observed in neurodegenerative disorders such as AD, MS and PD.

## 5. Key Molecules that Support Complement-Dependent Inhibitory Synapse Elimination by Microglia

### 5.1. ATP

As mentioned above, the complement cascades might be enhanced in the epileptic brain. The complement molecules C1q and C3 serve as “eat-me” signals that stimulate microglia to engulf synapses, which suggests that synapse elimination by microglia is promoted in the epileptic brain. However, it should be noted that microglia first need to contact or find the complement-tagged synapses to ultimately engulf them.

Accumulating studies have shown that ATP-dependent structural interactions between microglia and neurons are enhanced when neuronal activity is increased [80,81,82,83]. It has also been shown that ATP concentrations are elevated in acute brain slices prepared from mice 2 h after kainic acid-induced status epilepticus [84]. Furthermore, neuronal activity induction by such methods as microwave exposure of the rat somatosensory cortex or electrical stimulation of mouse hippocampal Schaffer collaterals increases ATP concentrations in recording buffer [85,86]. These results suggest that ATP release from neurons is accelerated in the epileptic brain. Microglia express many types of purinergic receptors, and the expression levels of these receptors are increased after epileptic seizures [21]. Therefore, it is plausible that ATP-mediated microglia–neuron interaction is promoted in epilepsy.

#### 5.1.1. ATP-Mediated Microglia–Neuron Interaction

In vivo live imaging of the brain and live imaging of acute brain slices using two-photon excitation microscopy have shown that microglial processes extend toward ATP injection sites [80,87]. This phenomenon is inhibited by knocking out P2Y12, a purinergic receptor that is specifically expressed by microglia in the brain [80]. Therefore, ATP has been considered a “find-me” signal for microglia to find active neurons. Neurons express the ATP channel pannexin1, which opens to release ATP in response to NMDA receptor activation [88,89,90]. In addition, when neuronal NMDA receptors are activated, surrounding microglia become hyperramified and extend their processes [83]. From these findings, it is widely believed that activated neurons secrete ATP and induce microglial process elongation. Astrocytes also release ATP in a KA-induced mouse model of SE and ATP from astrocytes could modulate neuronal activity [91,92]. Because astrocytic feet locate closely to synapses, forming tripartite synapses, it is possible that astrocytic ATP attracts microglial processes toward synapses. However, it has also been reported that the pannexin1 antibody CT-395 cannot suppress microglial dynamics in response to NMDA receptor activation, raising the possibility that there are factors other than ATP that serve as “find-me” signals [83].

#### 5.1.2. Engulfment of Inhibitory Synapses

Many previous studies have focused on ATP release from excitatory neurons. However, some inhibitory neurons express channels such as pannexin1, connexin26, connexin32 and connexin36, through which ATP can be released, raising the possibility that inhibitory neurons can also secrete ATP [93,94,95,96,97]. Some studies have indicated that not only excitatory neurons, but also inhibitory neurons, exhibit increased activity in the context of epilepsy. Muldoon et al. simultaneously recorded EEG and calcium signals in the hippocampal CA1 area in a pilocarpine-induced mouse model of mTLE [98]. They found that interictal spikes of GABAergic neurons were dominant in the CA1 and that their activity suppressed the excitation of excitatory CA1 pyramidal neurons. Peng and Houser also used a pilocarpine-induced mouse model of mTLE and found that dentate granule cells were activated 30 min after spontaneous seizures but exhibited control activity levels by 2 h after the seizures; in contrast, the increased activity of hilar GABAergic interneurons was maintained 2 to 4 h after the seizures [99]. Together, these results suggest that the activity of inhibitory neurons is maintained after epileptic seizures for a certain period, implying sustained release of ATP and possible induction of microglial process extension.

Studies on microglia-dependent synapse elimination have mostly focused on excitatory synapses or have not distinguished between excitatory and inhibitory synapses [12,71,72]. Few studies have investigated inhibitory synapse engulfment specifically; however, under some conditions, microglia wrap inhibitory presynapses with their processes and induce synaptic E/I imbalance by manipulating synaptic transmission in a process called synapse stripping. Trapp et al. observed synapse stripping in the cerebral cortices of rats that were subjected to the intracerebral administration of Bacillus Calmette-Guérin (BCG) bacteria [100]. This BCG injection models focal cortical inflammation in rats. After peripheral immune challenge by subcutaneous injection of BCG, microglial cell bodies are enlarged and located close to neuronal cell bodies, but the underlying molecular mechanisms remain unclear. As a result, the neuronal perikaryal areas occupied by presynaptic terminals were decreased in number in the BCG group compared to the control group. Most axosomatic synapses are inhibitory, so authors have argued that synapse stripping is an inhibitory synapse-specific phenomenon. In rodent models of inflammation induced by lipopolysaccharide (LPS) administration, microglial contacts to neurons are increased, and the neuronal perikaryal areas occupied by inhibitory presynaptic terminals are decreased in the cortex [101]. Furthermore, neuronal firing is increased in these models. Cerri et al. have suggested CCL2–CCR2 signaling as a mechanism by which LPS-induced inflammation enhances epileptic seizures via microglial activation [102]. They showed that after LPS administration to KA-induced epileptic mice, expression levels of CCL2 and CCR2 are increased in hippocampal neurons and microglia, respectively. Neuronal CCL2 has been suggested to induce microglia-dependent synaptic engulfment in the lymphocytic choriomeningitis virus-induced encephalitis model mice, but direct evidence is lacking [103]. Together, these findings indicate that inhibitory neurons with increased activity may enable microglia to find them by releasing “find-me” signals, including ATP, in the epileptic brain, which may ultimately result in the stripping and engulfment of inhibitory synapses in the context of epilepsy. A reduction in effective inhibitory synaptic transmission would tip the synaptic E/I balance toward excitation, promoting epileptogenesis and aggravating epileptic symptoms. Then, how can microglia strip synapses when astrocyte processes wrap presynapses and postsynapses by forming tripartite synapses? Plata et al. have shown that astrocytes decrease their morphological complexity (i.e., decreased number of branches) after pilocarpine-induced SE, so it is possible that the phenomena allow microglia to easily access synapses in the epileptic brain [104].

### 5.2. Progranulin

#### 5.2.1. Synaptic E/I Imbalance Induced by Progranulin Mutation

Recently, inhibitory specific synapse engulfment by microglia has been observed in mice lacking *Grn*, which codes progranulin [25]. Progranulin is a risk gene for frontotemporal lobar degeneration and decreases the inflammatory response mediated by microglia and macrophages [105,106,107]. In the brain, progranulin mRNA is expressed mainly by microglia, and *Grn* knockout increases the expression of complement molecules and induces the engulfment of synaptophysin by microglia [25]. Interestingly, although C1q tagging is increased in both excitatory and inhibitory synapses, only inhibitory synapse density is decreased. Furthermore, *Grn* knockout mice show increased frequencies of action potentials in the ventral thalamus, indicating synaptic E/I imbalance (Figure 1B). The mechanisms by which inhibitory synapses are selectively reduced remain unclear. The authors concluded that it is due to differences in the expression levels and distribution of complement receptors or other recognition molecules between excitatory and inhibitory synapses. Stephan et al. found that C1q protein levels were increased in the human and mouse brain parenchyma during normal aging. In the mouse hippocampus, all microglia were stained with an anti-C1q antibody [108]. They also showed that approximately 30% of GABAergic neurons were C1q positive and that all C1q positive neurons were GABAergic. From these results, we can also speculate that C1q, which is released from microglia, preferentially accumulates in inhibitory neurons.

#### 5.2.2. Progranulin in the Epileptic Brain

Progranulin has recently gained attention in epilepsy research. One study measured progranulin levels in cerebrospinal fluid taken from epileptic patients and found that they were slightly increased after status epilepticus [109]. The authors argued that progranulin was increased by a compensatory mechanism for neuronal hyperactivation because this molecule works as a nerve growth factor and suppresses inflammation, probably protecting damaged neurons and downregulating proinflammatory cytokines after seizures. A rodent study also showed that progranulin expression by microglia and macrophages was increased in the cortex, hippocampus and thalamus 48 h after pilocarpine-induced status epilepticus [110]. Another study reported that two patients suffering neuronal ceroid lipofuscinosis experienced recurrent generalized seizures and that they had progranulin gene mutations [111].

Overall, this evidence indicates that progranulin mutations may increase complement levels and induce the engulfment of inhibitory synapses by microglia, leading to synaptic E/I imbalance and epileptogenesis. Increases in progranulin levels after status epilepticus might act in a feedback inhibition system to suppress further synapse engulfment.

### 5.3. SV2A

Microglia have been suggested to engulf presynapses in animal models of inflammation and systemic lupus erythematosus (SLE)—both of which are risk factors for epilepsy [26,29]. However, it has not yet been revealed whether microglia are capable of selectively engulfing excitatory or inhibitory synapses in these conditions. Furthermore, it remains unclear whether microglia preferentially engulf postsynapses or presynapses (most studies have relied on postsynaptic markers to examine synapse engulfment by microglia): one report mentioned presynapse-specific engulfment, but another report showed decreased postsynaptic density without reference to postsynapse engulfment [26,29].

How is the synaptic E/I balance affected when microglia engulf both excitatory and inhibitory presynapses (Figure 1C)? The synaptic vesicle protein SV2, a presynaptic marker, has been suggested to be relevant to epilepsy. There are three subtypes of SV2 (SV2A, SV2B and SV2C), the expressing cell types and regions of which differ [112]. SV2A is found in all brain regions and is expressed by both glutamatergic and GABAergic neurons. However, SV2B is absent in the dentate gyrus and substantia nigra and is expressed by only glutamatergic neurons. Although deficits in brain development have not been reported after SV2B knockout, SV2A-knockout mice have epileptic seizures and die by 2 weeks of age [27]. In addition, CA1 pyramidal neurons in acute slices prepared from SV2A knockout mice show increased frequencies of spontaneous excitatory postsynaptic currents and decreased frequencies and amplitudes of spontaneous inhibitory postsynaptic currents [28]. These results suggest that deficits in the transmission of excitatory and inhibitory synapses together lead to synaptic E/I imbalance. It is possible that malfunction of inhibitory synapses is more prominent than that of excitatory synapses, because inhibitory synapses are located closer than excitatory synapses to the somas of recipient cells, which implies that inhibitory inputs are more robust and have larger effects on the activity of recipient cells [112]. SV2A is decreased in the anterior temporal cortices of patients with intractable TLE, and the extent is more severe in patients with hippocampal sclerosis than in those without [113,114].

Synapsin, another presynaptic protein, has also been suggested to be involved in epilepsy. There are three subtypes of synapsin (synapsin I, synapsin II and synapsin III) and the expressing cell types differ: synapsin I is mainly expressed by inhibitory neurons but synapsin II is expressed by excitatory neurons [115]. Mice with gene mutations in synapsin I and/or synapsin II exhibited spontaneous seizures from 2 months of age [116]. In the hippocampus of synapsin I/II/III knockout mice, increased glutamatergic and decreased GABAergic neurotransmission were observed before behavioral seizures occur [117]. Furthermore, synapsin I gene mutations were found in the patients with epilepsy and/or ASDs [118]. Given these findings, it is possible that when microglia engulf both excitatory and inhibitory synapses, inhibitory neurotransmission is severely decreased, leading to synaptic E/I imbalance and aggravation of epileptic seizures.

## 6. Conclusions

In this review, we have discussed the relationships between epilepsy and microglia-dependent synaptic pruning, suggesting that synaptic pruning could induce the development and aggravation of epilepsy. Animal models of inflammatory diseases accompanied by epileptic seizures show elevated expression levels of complement molecules and increased synapse engulfment by microglia. Future studies are necessary to determine which synapses (i.e., pre- or postsynapses and inhibitory or excitatory synapses) are specifically engulfed by microglia. However, as introduced in Section 5.3, even if both excitatory and inhibitory synapses are decreased in the same proportion, the E/I balance of the neural circuit might tip toward excitation. These results indicate that aberrant synaptic pruning by microglia induces synaptic E/I imbalance, leading to the development and aggravation of epilepsy. Additionally, mutations that result in inhibitory synapse-specific engulfment by microglia, such as progranulin mutation, might also contribute to epileptogenesis. We have further mentioned the possibility that not only excitatory but also inhibitory neurons release ATP, a “find-me” signal for microglial synapse engulfment, when inhibitory neurons are hyperactive under epileptic conditions. If activated inhibitory synapses are removed by microglia, synaptic E/I balance will result in excitatory dominant conditions, resulting in the exacerbation of epileptic symptoms. However, it remains unknown whether/how inhibitory synapses are specifically engulfed by microglia during epileptogenesis. To answer this question, the spatiotemporal relationships between cellular (both neuronal and microglial) activation and the expression of molecules that mediate synapse engulfment, such as complement molecules, must be clarified.
